# Age-related differences in sperm chromatin packaging and protein profiles based on SDS-PAGE in Bali bulls (*Bos javanicus*)

**DOI:** 10.5455/javar.2026.m1053

**Published:** 2026-06-22

**Authors:** Zulfi Nur Amrina Rosyada, Hikmayani Iskandar, Mirni Lamid, Mohammad Anam Al Arif, Widya Paramita Lokapirnasari, Rimayanti Rimayanti, Tulus Maulana, Vincentia Trisna Yoelinda, Siti Rani Ayuti, Amirul Faiz Mohd Azmi

**Affiliations:** 1 Division of Animal Husbandry, Animal Science, and Nutrition Research Group, Faculty of Veterinary Medicine, Universitas Airlangga, Surabaya, East Java 60115, Indonesia; 2 Research Center for Applied Zoology, National Research and Innovation Agency (BRIN), Cibinong, Bogor, West Java 16911, Indonesia; 3 Division of Veterinary Reproduction, Reproduction Engineering Research Group, Faculty of Veterinary Medicine, Universitas Airlangga, Surabaya, East Java 60115, Indonesia; 4 Department of Physiology, Faculty of Veterinary Medicine, Universitas Gadjah Mada, Yogyakarta 55281, Indonesia; 5 Doctoral Program of Veterinary Science, Faculty of Veterinary Medicine, Universitas Airlangga, Surabaya, East Java 60115, Indonesia; 6 Laboratory of Biochemistry, Faculty of Veterinary Medicine, Universitas Syiah Kuala, Jl. Tgk. Hasan Krueng Kalee No. 4, Kopelma Darussalam, Banda Aceh 23111, Aceh, Indonesia; 7 Department of Veterinary Preclinical Sciences, Faculty of Veterinary Medicine, Universiti Malaysia Kelantan, 16100 Pengkalan Chepa, Kelantan, Malaysia

**Keywords:** Bali cattle, chromatin integrity, food security, protamine, SDS-PAGE, sperm motility

## Abstract

**Objectives:** This study evaluated the effect of advancing age on sperm chromatin integrity and protamine status and also evaluated sperm protein profiles based on sodium dodecyl sulfate–polyacrylamide gel electrophoresis (SDS-PAGE) in Bali bulls (*Bos javanicus*). The study also examined the relationship between chromatin integrity parameters and sperm motility to provide insights into molecular determinants of sperm quality supporting sustainable cattle production and food security.

**Materials and Methods:** Eight Bali bulls maintained at an artificial insemination center were divided into two groups: bulls younger than 10 years (*n* = 4) and bulls older than 10 years (*n* = 4). Sperm motility parameters were evaluated using computer-assisted sperm analysis (CASA). Chromatin integrity was assessed through acrosome integrity, DNA fragmentation, and protamine status using microscopic staining techniques, including chromomycin A3 (CMA3). Sperm protein profiles were analyzed using SDS-PAGE followed by densitometric analysis with ImageJ. Statistical analysis was performed using independent sample *t*-tests and Pearson correlation.

**Results:** Bulls older than 10 years showed significantly higher progressive motility (*p* = 0.04) and protamine status (*p* = 0.03) than younger bulls, while acrosome integrity and DNA fragmentation were not significantly different. A strong positive relationship was observed between protamine status and progressive motility (R^2^ = 0.72, *p* = 0.007). SDS-PAGE revealed several protein bands, with the ~10–11 kDa band showing significantly higher intensity in older bulls (*p* = 0.007).

**Conclusions:** Advancing age in Bali bulls was associated with improved protamine-mediated chromatin packaging and higher progressive sperm motility. Integrating chromatin integrity assessment with sperm protein profiling may support more effective bull selection strategies and contribute to improved cattle reproduction and food security.

## 1. Introduction

Bali cattle (*Bos javanicus*) are an indigenous Indonesian breed widely recognized for their adaptability to tropical environments and relatively high reproductive efficiency, making them an important genetic resource for beef production in Southeast Asia [1]. The success of breeding programs and artificial insemination largely depends on the quality of spermatozoa produced by breeding bulls, which is commonly evaluated through parameters such as sperm motility, morphology, and viability. However, conventional spermatozoa analysis does not always accurately predict bull fertility because additional molecular factors may influence spermatozoa function and fertilization outcomes [2, 3].

Among the molecular determinants of spermatozoa quality, chromatin integrity has received increasing attention [3]. During spermiogenesis, histones are replaced by transition nuclear proteins and subsequently by protamines, resulting in highly condensed chromatin that protects paternal DNA during sperm transport and fertilization [4]. Protamines are arginine-rich nuclear proteins that tightly package sperm DNA, ensuring genomic stability and proper chromatin condensation in mature spermatozoa [4, 5]. In mammals, two main types of protamines have been identified, namely protamine 1 (PRM1) and protamine 2 (PRM2), although their relative expression and functional importance may vary among species, with PRM1 being the predominant form in many livestock species. PRM2 is synthesized as precursor proteins that undergo proteolytic processing to form mature protamines, whereas PRM1 is typically expressed in its mature form. The balance and proper processing of these protamines are essential for normal chromatin condensation and sperm function, and alterations in their expression or maturation may lead to defective chromatin packaging and reduced fertility [6]. According to Baharun et al. [7], alterations in protamine levels, including deficiency or imbalance, can compromise chromatin packaging, leading to DNA instability, morphological abnormalities in spermatozoa, and reduced fertility potential.

Several studies have reported that sperm chromatin integrity, including protamine status, is associated with semen quality and fertility in bulls. Recent research has shown that protamine deficiency may impair chromatin condensation and increase sperm DNA’s susceptibility to damage in bovine spermatozoa [7, 8]. Abnormalities in chromatin packaging are also associated with variations in sperm functional parameters such as motility and morphology in breeding bulls. Despite these findings, the relationship between chromatin packaging and sperm functional parameters remains complex and may vary depending on breed and physiological conditions [5].

In addition to chromatin organization, the sperm proteome plays a critical role in sperm function, fertilization, and early embryo development. According to Druart and de Graaf [9], changes in sperm protein composition may reflect alterations during spermatogenesis and can influence motility, membrane integrity, and fertilization capacity. In cattle, variations in sperm protein profiles have been reported in association with differences in semen quality and fertility potential [2]. Techniques such as SDS-PAGE have been widely used to characterize sperm protein patterns and identify potential biomarkers of sperm function [2, 10].

Bali bulls (*B. javanicus*) typically reach sexual maturity at approximately 2–3 years of age, although optimal reproductive performance is generally achieved at a slightly later stage. Based on reproductive development, bulls can be broadly categorized into young (post-pubertal), mature, and older age groups. Mature bulls are generally considered to be within the age range of approximately 4 to 10 years, during which semen quality and reproductive efficiency are optimal [1]. In practical breeding systems, particularly in artificial insemination centers, breeding males are typically selected within this mature age range due to their stable semen quality and reproductive performance. Bulls older than 10 years may still be reproductively active; however, age-related changes in sperm characteristics may begin to occur. Therefore, the age classification used in this study (younger than 10 years and older than 10 years) reflects a comparison between bulls within the optimal reproductive stage and those approaching later stages of reproductive life.

Age is another factor known to influence reproductive performance in breeding bulls. Kumaresan et al. [11] reported that age-related changes may affect semen quality, chromatin integrity, and spermatogenic processes, potentially altering the structural and molecular characteristics of spermatozoa. However, information regarding the combined effects of age on protamine status, chromatin integrity, and sperm protein composition in Bali bulls remains limited. Importantly, studies investigating the relationship between chromatin packaging status (CMA3-based), sperm chromatin integrity, and sperm protein profiles in indigenous cattle breeds remain scarce. Most previous studies have focused on conventional semen parameters or individual molecular markers without integrating chromatin packaging status with sperm protein characterization. Therefore, simultaneous evaluation of chromatin integrity and sperm protein profiles may provide more comprehensive insights into the molecular mechanisms underlying sperm quality in Bali bulls.

This study aimed to evaluate the effect of advancing age on sperm chromatin integrity and protamine status and to characterize sperm protein profiles using SDS-PAGE in Bali bulls. In addition, this study examined the relationship between chromatin integrity parameters and sperm motility to provide further insights into molecular factors influencing sperm quality in this important indigenous cattle breed.

## 2. Materials and Methods

### 2.1. Ethical approval

All procedures involving animals were reviewed and approved by the Health Research Ethical Clearance Commission, Faculty of Dental Medicine, Universitas Airlangga, Indonesia (Approval No. 0098/HRECC.FODM/I/2026). All experimental procedures were conducted in accordance with institutional guidelines for the care and use of animals in research.

### 2.2. Study location and experimental animals

The study was conducted at the Artificial Insemination Center (BIBD Pucak), Maros, South Sulawesi, Indonesia. A total of eight Bali bulls maintained under routine artificial insemination management were included in this study. The bulls were clinically healthy and had known reproductive histories. Based on age, the bulls were divided into two groups: bulls younger than 10 years (*n* = 4) and bulls older than 10 years (*n* = 4). The age classification followed standard grouping practices at the artificial insemination center, using a 10-year threshold to distinguish younger and mature bulls. All animals were housed under similar management conditions and received standard feeding consisting of forage and concentrate according to the breeding center management system. Age has been reported to influence semen quality and reproductive performance in breeding bulls [12].

### 2.3. Semen collection and sample preparation

Semen samples were collected using an artificial vagina according to standard semen collection procedures routinely applied in breeding bulls [12]. Each bull was collected three times to obtain biological replicates, with approximately 7-day intervals between collections following the routine schedule of the artificial insemination center. Each ejaculate was analyzed individually for sperm motility and chromatin integrity parameters to represent biological replicates. All analyses were performed using cryopreserved semen samples following thawing (post-thaw). Semen samples were collected and cryopreserved according to standard procedures routinely applied at the artificial insemination center. Frozen semen samples used in this study were obtained from the artificial insemination center and cryopreserved according to standard protocols routinely applied at the facility. Briefly, semen was diluted with a commercial extender (AndroMed^®^), gradually cooled, equilibrated at low temperature, filled into straws, and subsequently cryopreserved in liquid nitrogen. Frozen semen samples were thawed in a water bath at 37°C for 30 sec according to the protocol described by Rosyada et al. [2]. All subsequent evaluations, including sperm motility, chromatin integrity, and protein analysis, were conducted using post-thaw semen samples. The samples were then washed three consecutive times with phosphate-buffered saline (PBS) to remove cryoprotectants and extender residues, followed by centrifugation at 1,800 *× g* for 5–10 min. The resulting sperm pellets were subsequently used for chromatin integrity assessment and protein extraction. For sperm protein analysis, protein extraction was performed at the individual bull level, and one representative sample per bull was used for SDS-PAGE analysis. Therefore, each lane in the gel represents one bull rather than individual ejaculates.

### 2.4. Computer-assisted sperm analysis (CASA)

Sperm motility and kinematic parameters were evaluated using a CASA system following previously described procedures with minor modifications [13, 14]. Briefly, semen samples were diluted with semen extenders to obtain an appropriate sperm concentration for analysis. A 5 μl aliquot of diluted semen was placed on a pre-warmed microscope slide (38°C) and covered with an 18 mm × 18 mm coverslip. The samples were examined using an Axio Scope A1 microscope (Carl Zeiss, Oberkochen, Germany) equipped with a phase-contrast objective at 200x magnification, connected to Sperm Vision version 3.7.8 software (Minitube, Tiefenbach, Germany). The microscope stage temperature was maintained at 37–38°C during analysis to preserve sperm motility. Images from eight microscopic fields were captured, and motility parameters were calculated from approximately 1000 spermatozoa per sample. The CASA system was used to evaluate several sperm motility parameters, including total motility (TM, %), progressive motility (PM, %), average path velocity (VAP, μm/sec), curvilinear velocity (VCL, μm/sec), and velocity straight-line (VSL, μm/sec). CASA-based analysis provides objective and quantitative evaluation of sperm motility characteristics and has been widely applied in bovine reproductive studies [13, 14].

### 2.5. Assessment of sperm structural and chromatin integrity

Sperm structural and chromatin integrity were evaluated through the assessment of acrosome integrity, DNA integrity, and chromatin packaging status using fluorescence-based staining techniques. Semen smears were prepared on clean glass slides, air-dried, and subjected to specific staining procedures. Acrosome integrity was assessed using fluorescein isothiocyanate-conjugated peanut agglutinin (FITC-PNA) combined with propidium iodide (PI). Spermatozoa exhibiting bright green fluorescence in the acrosomal region were classified as having intact acrosomes, whereas those lacking acrosomal fluorescence were considered to have damaged acrosomes. DNA integrity was evaluated using the sperm chromatin dispersion (SCD) method with a commercial Halomax^®^ kit (Halotech DNA S.L., Spain) according to the manufacturer’s instructions. Briefly, semen samples were embedded in agarose on pre-treated slides, lysed to remove nuclear proteins, and subjected to controlled denaturation conditions to allow DNA dispersion. Following processing, spermatozoa were stained with a DNA-binding fluorescent dye and examined under a fluorescence microscope at 1000x magnification. The assessment was based on the presence and size of chromatin dispersion halos. Spermatozoa with intact DNA exhibited compact nuclei with minimal or no halo formation, whereas spermatozoa with fragmented DNA displayed dispersed chromatin characterized by large halos surrounding the nuclear core. At least 200 spermatozoa per sample were evaluated to determine the percentage of sperm with intact DNA, following previously described criteria for SCD-based DNA fragmentation assessment. DNA integrity (%) was expressed as the percentage of spermatozoa with intact DNA, which inversely reflects DNA fragmentation levels assessed by the SCD method [15]. In addition to these parameters, chromatin packaging and protamine deficiency were further evaluated using CMA3 staining, which is described in a separate section below.

### 2.6. CMA3-based assessment of chromatin packaging and protamine deficiency

Chromatin packaging status was assessed using the Chromomycin A3 (CMA3) staining method, which indirectly reflects protamine deficiency, as previously described by Carreira et al. [16] with minor modifications. Frozen-thawed semen samples were smeared onto clean glass slides and air-dried. The slides were then fixed in methanol/glacial acetic acid (3:1) for 5 min at 4°C. Subsequently, the slides were incubated with CMA3 staining solution (0.25 mg/ml CMA3 in McIlvaine’s buffer, pH 7.0, supplemented with 10 mM MgCl_2_) for 20 min in a dark chamber. After staining, the slides were rinsed with McIlvaine’s buffer and allowed to air-dry. The stained samples were examined under a fluorescence microscope at 1000x magnification, and representative images were captured to illustrate CMA3 staining patterns. A total of 500 spermatozoa per sample were evaluated to ensure robust quantitative assessment. Spermatozoa exhibiting bright green fluorescence in the sperm head were classified as CMA3-positive, indicating reduced chromatin condensation and potential protamine deficiency, whereas spermatozoa showing faint fluorescence were considered CMA3-negative, indicating normal chromatin condensation. The percentage of sperm with normal chromatin packaging was calculated for each sample based on CMA3 staining patterns. CMA3 staining is widely used as an indirect method to evaluate sperm chromatin packaging and protamine deficiency in reproductive studies [16, 17]. It should be noted that CMA3 staining provides an indirect assessment of chromatin packaging and protamine deficiency based on chromatin accessibility and does not directly quantify protamine protein levels.

### 2.7. Sperm protein extraction

Total sperm proteins were extracted from sperm pellets using PRO-PREP^™^ protein extraction solution (iNtRON Biotechnology, Seongnam, Korea) according to the manufacturer’s instructions. Briefly, sperm pellets were lysed using an extraction buffer and incubated on ice to facilitate protein solubilization. The lysate was then centrifuged to remove cellular debris, and the supernatant containing soluble proteins was collected. Protein concentration was determined using the bicinchoninic acid (BCA) protein assay to ensure equal protein loading prior to electrophoresis, as commonly applied in sperm proteomic studies [18].

### 2.8. SDS-PAGE analysis of sperm proteins

Sperm protein profiles were analyzed using SDS-PAGE. Protein concentrations were first determined using the BCA assay, and equal amounts of protein were loaded for each sample. The protein extracts were then mixed with loading buffer and denatured prior to electrophoresis. Protein separation was performed using a 12% polyacrylamide precast gel (ExpressPlus^™^, M01210; Genscript Biotech Corp., Hong Kong) under denaturing conditions at a constant voltage of 140 V and 75 mA for approximately 55 min. A pre-stained broad-range protein ladder (M00624, Genscript Biotech Corp., Hong Kong) with a molecular weight range of 5–270 kDa was included in each run to facilitate molecular weight determination. Following electrophoresis, gels were stained with Coomassie Brilliant Blue (CBB) to visualize protein bands. SDS-PAGE is widely used to characterize sperm protein profiles and identify proteins associated with sperm function and fertility [7].

### 2.9. Densitometric analysis of protein bands

The relative intensity of protein bands was quantified using ImageJ (National Institutes of Health, Bethesda, MD, USA). Gel images were converted to grayscale, and densitometric analysis was performed using the gel analysis tool. The peak area corresponding to each selected protein band was measured, and relative protein intensity was calculated by normalizing band intensity to the total lane intensity. The resulting values were expressed as arbitrary units (AU). Quantitative densitometry allows comparison of protein abundance among samples and provides a reliable approach to evaluate differences in protein expression levels [2].

### 2.10. Statistical analysis

Data normality was assessed using the Shapiro–Wilk test. Differences between groups were analyzed using an independent sample t-test. The relationship between CMA3 and semen parameters was evaluated using Pearson correlation analysis. All statistical analyses were performed using SPSS version 25.0 (IBM Corp., Armonk, NY, USA), with *p* < 0.05 considered statistically significant. Data were presented as the mean ± standard error of the mean (SEM). Protein band intensity obtained from densitometric analysis was expressed in arbitrary units (AU) and presented descriptively.

## 3. Results

### 3.1. CASA sperm motility characteristics

Sperm motility parameters measured using CASA were presented in Table 1. Overall, bulls older than 10 years tended to show higher values for several motility parameters compared with bulls younger than 10 years. Total motility was numerically higher in bulls older than 10 years (48.12 ± 3.79%) compared with bulls younger than 10 years (44.41 ± 1.75%), although the difference was not statistically significant (*p* = 0.12). Similarly, velocity-related parameters, including VAP (75.59 ± 13.97 vs. 70.12 ± 2.61 µm/sec), VCL (108.76 ± 21.64 vs. 98.17 ± 3.14 µm/sec), and VSL (55.44 ± 14.21 vs. 49.79 ± 2.73 µm/sec), were numerically higher in bulls older than 10 years compared with younger bulls; however, these differences were not statistically significant (*p* > 0.05). However, progressive motility differed significantly between the two age groups. Bulls older than 10 years showed significantly higher progressive motility (39.53 ± 4.56%) compared with younger bulls (33.10 ± 2.09%) (*p* = 0.04).

**Table 1.** CASA sperm motility parameters in Bali bulls.

Parameter	<10 years (*n* = 4) (mean ± SD)	>10 years (*n* = 4) (mean ± SD)	*p*-value
Total motility (%)	44.41 ± 1.75	48.12 ± 3.79	0.12
Progressive motility (%)	33.10 ± 2.09a	39.53 ± 4.56b	0.04*
VAP (µm/sec)	70.12 ± 2.61	75.59 ± 13.97	0.47
VCL (µm/sec)	98.17 ± 3.14	108.76 ± 21.64	0.37
VSL (µm/sec)	49.79 ± 2.73	55.44 ± 14.21	0.46

**Note:** Values were presented as mean ± standard deviation (SD). Different superscripts within the same row indicate significant differences between age groups (*p* < 0.05). CASA, computer-assisted sperm analysis; VAP, velocity of the average path; VCL, curvilinear velocity; VSL, straight-line velocity.

### 3.2. Sperm structural integrity and chromatin packaging status

**Figure 1.**
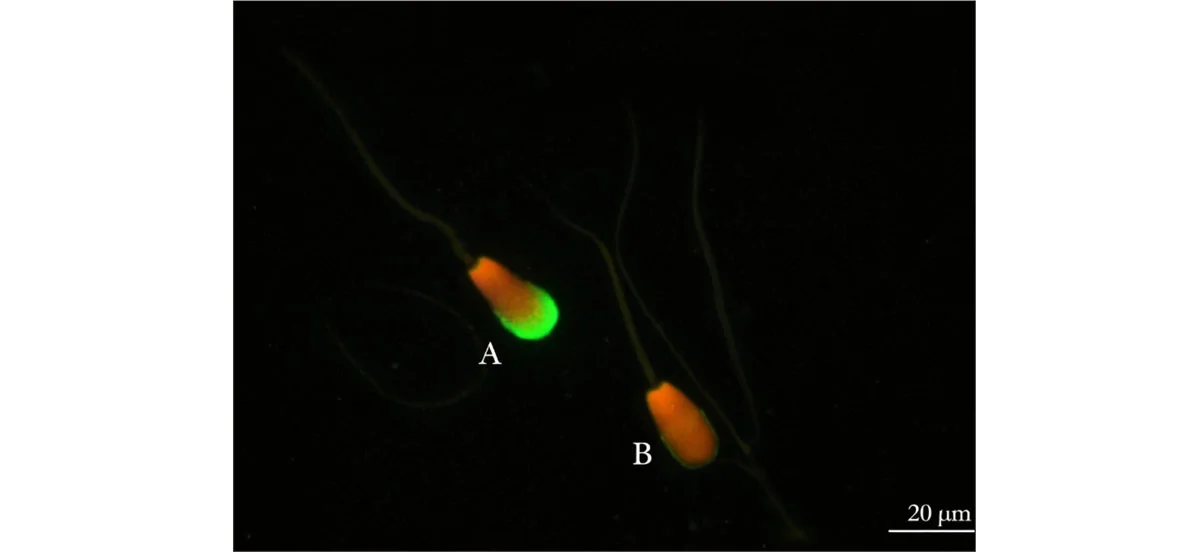
Representative fluorescence microscopy images of sperm acrosome integrity in Bali bulls. Spermatozoa exhibiting bright green fluorescence in the acrosomal region (A) were classified as intact acrosomes, whereas spermatozoa lacking green fluorescence (B) were considered to have damaged acrosomes.

**Figure 2.**
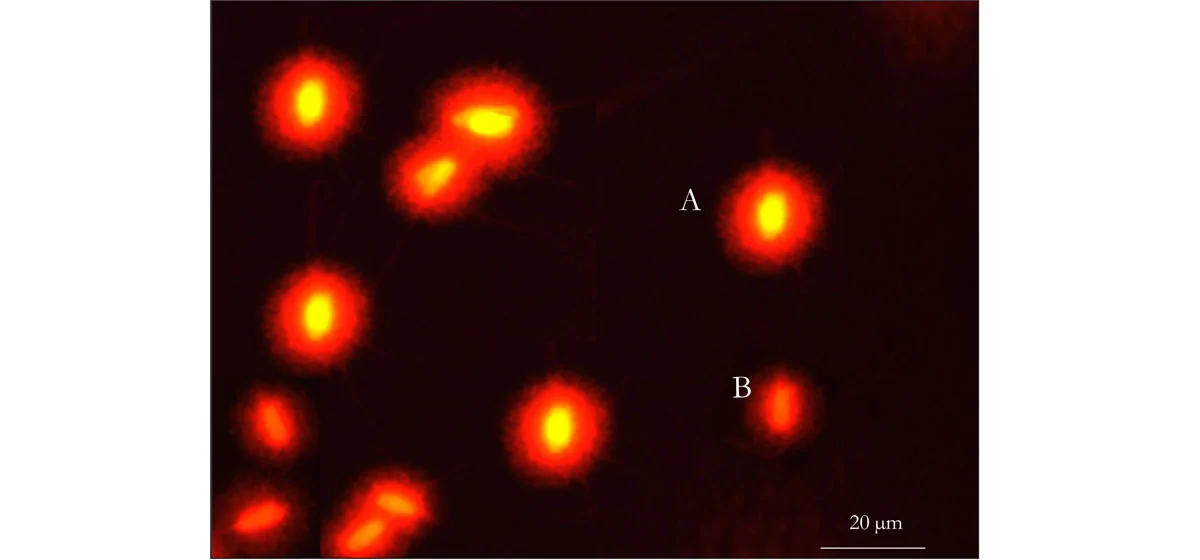
Representative fluorescence microscopy images of sperm DNA integrity in Bali bulls. Spermatozoa with fragmented DNA (A) showing dispersed chromatin characterized by a large halo surrounding the nuclear core. Spermatozoa with intact DNA (B) exhibiting compact nuclei with minimal or no halo formation.

**Figure 3.**
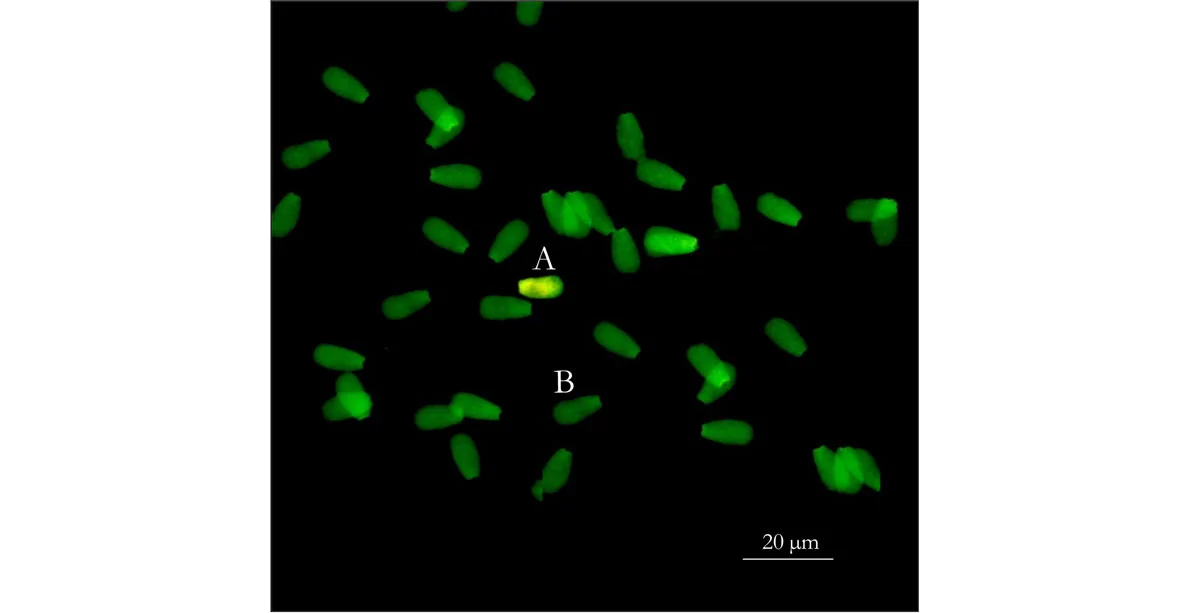
Representative CMA3 staining of spermatozoa in Bali bulls. Spermatozoa exhibiting bright green fluorescence in the sperm head (CMA3-positive) (A) indicates reduced chromatin condensation and potential protamine deficiency, whereas spermatozoa showing faint green fluorescence (CMA3-negative) (B) indicates normal chromatin packaging.

Sperm structural and chromatin-related parameters, including acrosome integrity, DNA integrity, and chromatin packaging assessed by CMA3 staining, were analyzed. Representative fluorescence images of acrosome integrity, sperm DNA integrity, and CMA3 staining were presented in Figures 1, 2, and 3, respectively, illustrating the fluorescence patterns used for classification. The results were summarized in Table 2. Acrosome integrity showed very similar values between the two age groups, with bulls younger than 10 years exhibiting 88.43 ± 3.90%, and bulls older than 10 years showing 88.34 ± 2.07%, with no significant difference (*p* = 0.96). Likewise, DNA integrity levels (expressed as the percentage of spermatozoa with intact DNA based on SCD analysis) were comparable between groups. Bulls younger than 10 years had 98.10 ± 0.68%, while bulls older than 10 years showed 98.55 ± 0.40%, indicating no statistically significant difference (*p* = 0.30). In contrast, chromatin packaging status (CMA3-based) differed significantly between age groups. Bulls older than 10 years demonstrated higher CMA3-negative sperm percentages (98.05 ± 1.67%) compared with younger bulls (95.10 ± 1.50%) (*p* = 0.03), indicating improved chromatin efficiency. This finding suggests improved chromatin packaging and a reduced likelihood of protamine deficiency in older bulls. The CMA3-based assessment provides an indirect measure of chromatin packaging and protamine deficiency, and the quantitative evaluation was based on scoring a large number of spermatozoa per sample to ensure reliability.

**Table 2.** Chromatin integrity parameters in Bali bulls.

Parameter	< 10 years (*n* = 4) (mean ± SD)	> 10 years (*n* = 4) (mean ± SD)	*p*-value
Acrosome integrity (%)	88.43 ± 3.90a	88.34 ± 2.077a	0.96
DNA integrity (%)	98.10 ± 0.68a	98.55 ± 0.40a	0.30
Protamine status (%)	95.10 ± 1.50a	98.05 ± 1.67b	0.03*

**Note:** Values are expressed as mean ± standard deviation (SD). Values were also presented for bulls younger than 10 years (*n* = 4) and older than 10 years (*n* = 4). Different superscripts within the same row indicate significant differences between age groups (*p* < 0.05). DNA integrity (%) represents the percentage of spermatozoa exhibiting intact DNA based on sperm chromatin dispersion (SCD) analysis using the Halomax^®^ method.

### 3.3. Relationship between chromatin integrity and sperm motility

**Figure 4.**
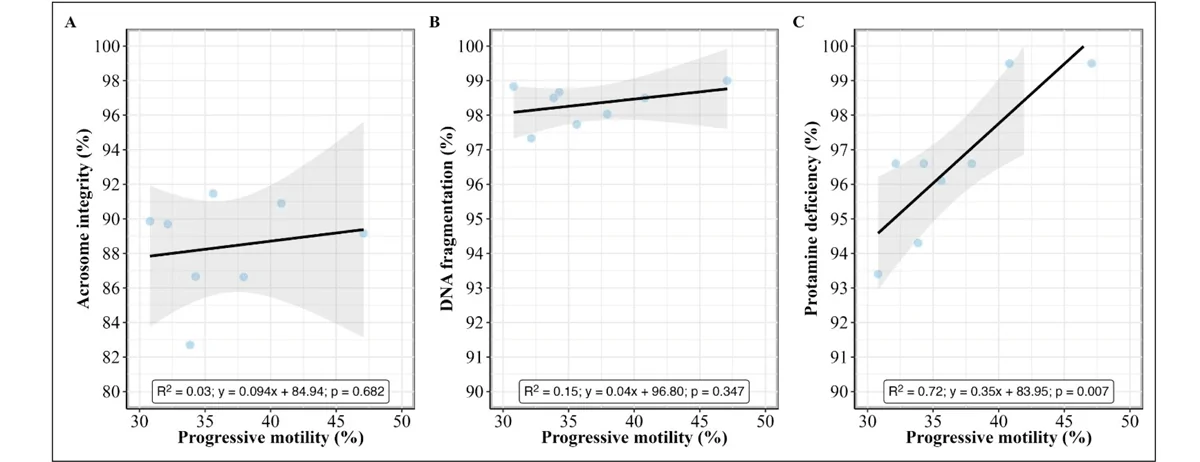
Relationship between progressive motility and chromatin integrity parameters. Panel A- Acrosome integrity vs. progressive motility; Panel B- DNA fragmentation vs. progressive motility; Panel C- Protamine status vs. progressive motility. Data points represent pooled samples from both age groups.

The relationship between progressive sperm motility and chromatin integrity parameters was further evaluated using linear regression analysis (Figure 4). Acrosome integrity showed a very weak relationship with progressive motility (R^2^ = 0.03, *p* = 0.682). Similarly, DNA fragmentation showed a weak and non-significant association with progressive motility (R^2^ = 0.15, *p* = 0.347). In contrast, chromatin packaging status (CMA3-based) exhibited a statistically significant positive association with progressive motility (R^2^ = 0.72, *p* = 0.007).

### 3.4. Sperm protein profiles revealed by SDS-PAGE

**Figure 5.**
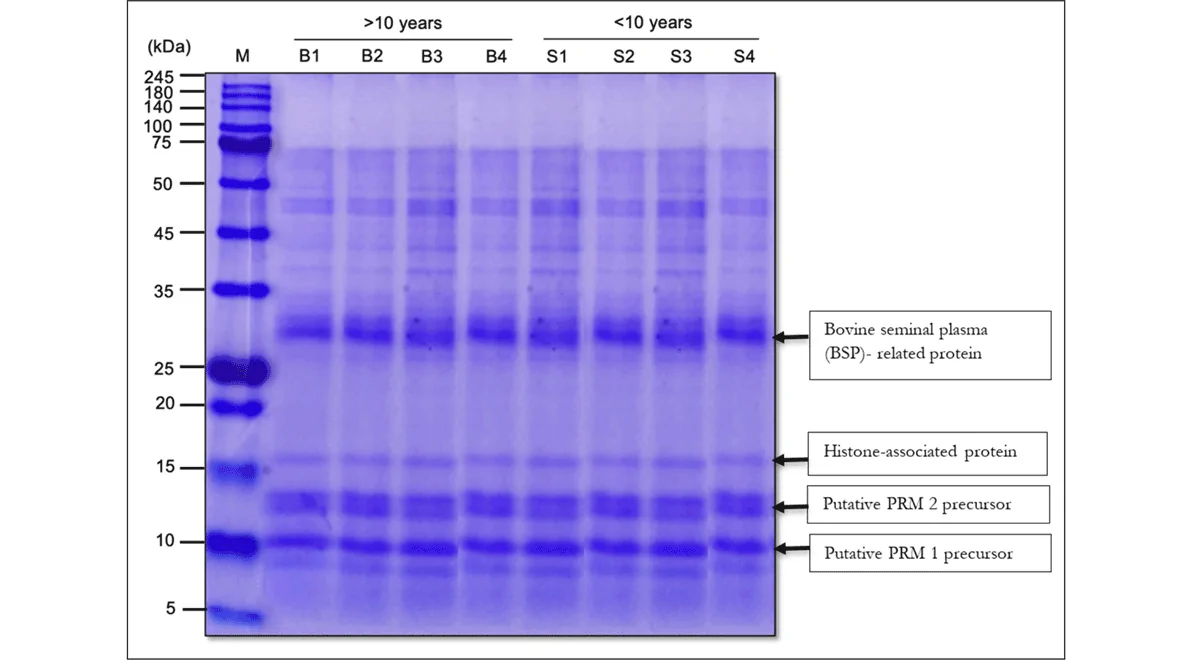
SDS-PAGE profile of sperm proteins in Bali bulls. Lane order reflects the original sample loading sequence during SDS-PAGE analysis. Selected protein bands of interest are indicated on the right side with their approximate molecular weights. M, molecular weight marker; B1–B4, bulls older than 10 years; S1–S4, bulls younger than 10 years.

The sperm protein profiles of Bali bulls were analyzed using SDS-PAGE, and the electrophoretic pattern is shown in Figure 5. The gel revealed multiple protein bands distributed across a molecular weight range of approximately 5–75 kDa. Several prominent protein bands were consistently observed across samples, particularly in the regions of approximately 10–11 kDa, 12 kDa, 16 kDa, and 30 kDa. Although the general band pattern appeared similar between age groups, variations in band intensity were observed between bulls older than 10 years and those younger than 10 years, suggesting potential age-related differences in sperm protein abundance based on electrophoretic profiling (Table 3). The observed differences were based on band intensity variations and do not indicate definitive protein identification.

**Table 3.** Mean relative intensity of sperm protein bands between age groups.

Protein band	> 10 years (mean AU ± SD)	< 10 years (mean AU ± SD)	*p*-value	Possible protein identity
~10–11 kDa	3.28 ± 1.22a	1.66 ± 0.93b	0.007*	Protamine 1 (PRM1) [19]
12 kDa	1.15 ± 0.20	1.67 ± 0.92	0.32	Protamine 2 (PRM2) [20]
16 kDa	2.80 ± 1.22	2.19 ± 1.03	0.49	Histone-associated protein [21, 22]
30 kDa	2.70 ± 2.67	2.76 ± 0.71	0.89	Bovine seminal plasma (BSP)-related protein [23]

**Note:** Values are expressed as mean relative intensity (arbitrary units, AU) ± standard deviation (SD). Relative protein intensity was obtained from densitometric analysis using ImageJ software. Different superscripts within the same row indicate significant differences between age groups (*p* < 0.05). Molecular weights of protein bands were estimated based on comparison with a pre-stained protein ladder.

### 3.5. Densitometric analysis of protein bands

The relative intensity of selected protein bands was further quantified through densitometric analysis (Table 3). Among the evaluated bands, the ~10–11 kDa protein band showed a significant difference between age groups. Bulls older than 10 years exhibited significantly higher relative intensity (3.28 ± 1.22 AU) compared with younger bulls (1.66 ± 0.93 AU) (*p* = 0.007). In contrast, the intensity of the 12 kDa, 16 kDa, and 30 kDa bands did not differ significantly between age groups (*p* > 0.05). These findings suggest that specific sperm proteins within the low molecular weight range may vary with age in Bali bulls.

## 4. Discussion

The present study investigated the relationship between age, sperm chromatin integrity, and sperm protein profiles in Bali bulls (*Bos javanicus*). The results indicated that bulls older than 10 years exhibited significantly higher progressive motility and protamine status compared with younger bulls, as shown in Tables 1 and 2. In addition, SDS-PAGE analysis revealed higher relative intensity of the ~10–11 kDa protein band in older bulls (*p* = 0.007), suggesting potential age-related differences in sperm protein abundance based on electrophoretic profiling (Table 3). Although the identity of this protein band was not determined in this study, the observed variation may be associated with differences in sperm chromatin organization or protamine packaging between the age groups.

Computer-assisted sperm analysis revealed that progressive motility was significantly higher in bulls older than 10 years (39.53 ± 4.56%) compared with younger bulls (33.10 ± 2.09%) (*p* = 0.04), as presented in Table 1. In contrast, total motility and velocity-related parameters, including VAP, VCL, and VSL, did not differ significantly between the two age groups (*p* > 0.05). Amann and Waberski [24] and Hossain et al. [25] reported that CASA-based analysis provides an objective and reproducible approach for evaluating sperm kinematics and has become a standard technique in livestock reproduction studies. Progressive motility is particularly important because it reflects the ability of spermatozoa to move efficiently toward the oocyte during fertilization. The higher progressive motility observed in older bulls may be associated with physiological maturity of the reproductive system and stabilization of spermatogenic and epididymal functions once bulls reach full reproductive maturity. Consistent with the present findings, Kumaresan et al. [11] reported that semen quality parameters in breeding bulls may improve with physiological maturity due to stabilization of spermatogenic processes and epididymal maturation. Similarly, Abah et al. [26] observed that semen characteristics may change with age, with mature animals often showing improved motility before reproductive performance eventually declines at advanced ages.

In contrast to the differences observed in sperm motility parameters, sperm structural integrity indicators, including acrosome integrity and DNA fragmentation as a marker of chromatin integrity, were comparable between the two age groups (Table 2). Both indicators showed similar values that were almost identical between younger and older bulls, suggesting that age did not markedly affect these aspects of sperm structural activity. Chromatin integrity is widely recognized as an important determinant of bull fertility because proper chromatin packaging ensures stable transmission of paternal genetic material to the embryo [27]. The tight packaging of sperm DNA by protamines contributes to protecting chromatin from oxidative stress and environmental damage during sperm transport within the female reproductive tract [28].

The absence of significant differences in DNA fragmentation between age groups in the present study may indicate that spermatogenic processes and chromatin protection mechanisms remain relatively stable in Bali bulls maintained under controlled breeding management conditions. Similar observations have been reported in bovine reproduction studies, where sperm DNA fragmentation levels in spermatozoa were generally low and remained relatively stable under normal physiological conditions and appropriate breeding management practices [29, 30, 31]. Kumaresan et al. [11] reported that efficient chromatin condensation and stable spermatogenesis contribute to maintaining DNA integrity in breeding bulls. Taken together, these findings suggest that the chromatin stabilization mechanisms that protect sperm DNA integrity may preserve the sperm in Bali bulls across the evaluated age range.

However, the present study revealed a significant difference in chromatin packaging status (CMA3-based) between age groups. As shown in Table 2, bulls older than 10 years exhibited a higher percentage of sperm with normal chromatin packaging (98.05 ± 1.67%) compared with younger bulls (95.10 ± 1.50%) (*p* = 0.03). Therefore, the observed differences likely reflect variations in chromatin packaging efficiency rather than direct changes in protamine protein abundance. It should be emphasized that CMA3 staining does not directly measure protamine protein levels but reflects chromatin accessibility and packaging status. Therefore, the observed differences likely represent variations in chromatin packaging efficiency rather than absolute changes in protamine abundance. According to Nagaki et al. [28] and Moritz and Hammoud [32], protamines are small arginine-rich nuclear proteins that replace histones during the final stages of spermiogenesis, resulting in highly condensed chromatin in mature spermatozoa. Proper protamine incorporation plays a crucial role in maintaining genomic stability and protecting sperm DNA from damage. Previous studies have shown that abnormal protamine expression or protamine deficiency can disrupt chromatin condensation and is associated with reduced male fertility in several mammalian species [33]. It should be noted that CMA3 staining provides an indirect assessment of protamine deficiency through chromatin accessibility and does not directly quantify protamine protein levels. Therefore, the results should be interpreted in the context of chromatin packaging rather than absolute protamine content.

The regression analysis further demonstrated a strong positive relationship between protamine status and progressive sperm motility (R^2^ = 0.72, *p* = 0.007), as illustrated in Figure 4. In contrast, acrosome integrity (R^2^ = 0.03, *p* = 0.682) and DNA fragmentation (R^2^ = 0.15, *p* = 0.347) showed weak and non-significant relationships with progressive motility. These results suggest that chromatin packaging mediated by protamines may show a stronger association with sperm motility compared with other chromatin integrity parameters in Bali bulls. Silva et al. [34] and Zhou et al. [35] reported that abnormalities in chromatin condensation may compromise sperm structural stability and increase susceptibility to DNA damage, which may ultimately impair sperm motility and fertilization capacity. Likewise, Kumaresan et al. [11] reported that sperm DNA integrity and chromatin packaging are closely associated with male fertility in farm animals.

The SDS-PAGE analysis revealed several protein bands within the molecular weight range of approximately 5–75 kDa (Figure 5). Among these proteins, the ~10–11 kDa band showed significantly higher intensity in bulls older than 10 years (3.28 ± 1.22 AU) compared with younger bulls (1.66 ± 0.93 AU) (*p* = 0.007), as shown in Table 3. Based on molecular weight estimation, this band may correspond to proteins in the low molecular weight range, including precursor forms of protamine 1 (PRM1), a major sperm nuclear protein responsible for chromatin condensation [3, 5, 19]. However, this assignment remains tentative and requires confirmation using protein-specific identification techniques. It should also be noted that protamines are small, highly basic proteins that may not be optimally resolved using standard SDS-PAGE conditions. Under conventional SDS-PAGE conditions, mature protamines are generally not resolved, and therefore, the detected bands are more likely to represent precursor forms. Recent evidence suggests that the accumulation of protamine precursor forms may reflect incomplete or altered protamine processing rather than improved chromatin condensation [36]. Therefore, increased abundance of these low molecular weight bands should be interpreted with caution, as they may indicate differences in protamine maturation efficiency rather than a direct improvement in sperm chromatin quality. Therefore, increased abundance of these low molecular weight bands should be interpreted with caution, as they may indicate differences in protamine maturation efficiency rather than a direct improvement in sperm chromatin quality. Definitive identification would require alternative approaches such as acid-urea PAGE, Western blotting, or mass spectrometry. SDS-PAGE analysis in this study was intended to provide a comparative overview of sperm protein profiles rather than definitive protein identification. The increased abundance of this protein in older bulls is consistent with the observed differences in chromatin packaging (CMA3-based) observed in the chromatin integrity analysis.

Another protein band detected in this study was located at approximately 12 kDa, which may correspond to precursor forms of protamine 2 (PRM2). Hamilton et al. [20] demonstrated that PRM2 can be detected in bovine spermatozoa and testicular tissue, although its abundance is generally lower than that of PRM1. In the present study, the relative intensity of this band did not differ significantly between age groups (*p* = 0.32), as shown in Table 3, suggesting that PRM2 expression may not be strongly influenced by age within the evaluated range.

The ~16 kDa protein band detected in the electrophoretic profile may represent histone-associated proteins involved in chromatin organization during spermatogenesis. During sperm development, histones are gradually replaced by protamines to achieve a highly condensed chromatin structure. Riaz et al. [21] and Karam and Moralo [22] described that histone or histone-replacement proteins play an essential role in chromatin remodeling processes during gamete differentiation. Meanwhile, the ~30 kDa band observed in the present study may correspond to bovine seminal plasma proteins (BSP), which are known to interact with sperm membranes and regulate capacitation and fertilization processes. Anwar et al. [23] reported that proteins within this molecular weight range are commonly detected in bovine seminal plasma and may influence sperm function and fertility.

Overall, the findings of the present study suggest that sperm chromatin packaging and proteins within the low molecular weight range may contribute to age-related differences in sperm motility in Bali bulls. The integration of CASA motility analysis, chromatin integrity assessment, and sperm protein profiling provides complementary insights into the molecular mechanisms underlying sperm quality in this indigenous cattle breed. Bali cattle represent an important indigenous genetic resource widely adapted to tropical environments in Southeast Asia. Previous studies have highlighted their ability to maintain reproductive performance under challenging environmental conditions and low-input production systems. Understanding the molecular characteristics of sperm quality in Bali bulls is therefore essential for improving breeding management and artificial insemination programs aimed at conserving and enhancing the productivity of this valuable indigenous cattle breed.

Despite these findings, several considerations should be taken into account when interpreting the results of this study. The present work focused on integrating conventional semen evaluation with chromatin integrity assessment and SDS-PAGE–based protein profiling to explore molecular characteristics of sperm quality in Bali bulls. While this approach provides valuable insights into the association between protamine-related proteins, chromatin integrity, and sperm motility, further studies combining these parameters with additional molecular or proteomic analyses may provide a more comprehensive understanding of sperm functional mechanisms.

The present study highlights the importance of protamine-mediated chromatin remodeling in maintaining sperm function in Bali bulls. Efficient chromatin condensation through proper protamine incorporation appears to be associated with improved progressive motility, suggesting that chromatin packaging may serve as a potential molecular indicator of sperm quality in breeding bulls.

## 5. Conclusions

This study demonstrated that advancing age influences several molecular and functional aspects of sperm quality in Bali bulls. Bulls older than 10 years exhibited significantly higher progressive sperm motility and improved chromatin packaging status (CMA3-based) compared with younger bulls, indicating more efficient chromatin packaging in mature animals. In addition, densitometric analysis of SDS-PAGE profiles revealed a significantly higher intensity of the ~10–11 kDa protein band, which may correspond to proteins within the low molecular weight range, including PRM1, based on molecular weight similarity reported in previous studies, in older bulls. However, this interpretation remains putative, as definitive protein identification was not performed. A strong positive relationship between protamine status and progressive motility further suggests that proper chromatin condensation plays an important role in maintaining sperm functional competence. These findings highlight the importance of integrating chromatin integrity evaluation with sperm protein profiling to better understand molecular determinants of sperm quality in indigenous cattle breeds. Such information may contribute to improving bull selection strategies and reproductive management in artificial insemination programs. Future studies combining chromatin integrity assays with advanced proteomic or molecular approaches are recommended to further elucidate the functional roles of sperm proteins associated with fertility in Bali bulls.

## Data Availability

The data presented in this study are available from the corresponding author upon reasonable request.

## List of abbreviations

AU, arbitrary unit; BCA, bicinchoninic acid; BSP, bovine seminal plasma protein; CASA, computer-assisted sperm analysis; CBB, Coomassie Brilliant Blue; CMA3, chromomycin A3; DNA, deoxyribonucleic acid; PBS, phosphate-buffered saline; PM, progressive motility; PRM1, protamine 1; PRM2, protamine 2; SDS-PAGE, sodium dodecyl sulfate–polyacrylamide gel electrophoresis; SEM, standard error of the mean; TM, total motility; VAP, velocity of the average path; VCL, curvilinear velocity; VSL, straight-line velocity; µm/sec, micrometer per second; mg/ml, milligram per milliliter; mM, millimolar; mm, millimeter; min, minutes; sec, seconds; kDa, kilodalton; V, volt; mA, milliampere; *×g*, relative centrifugal force.
